# Cooperating with Different Types of Strangers: The Influence of Guanxi Perception, Trust, and Responsibility

**DOI:** 10.3390/bs13060473

**Published:** 2023-06-05

**Authors:** Haoxin Liu, Tulips Yiwen Wang, Allan B. I. Bernardo, Heyong Shen

**Affiliations:** 1 Mental Health Education Centre, Jinan University, Guangzhou 510632, China; 2 Institute of Analytical Psychology, City University of Macau, Taipa 999078, Macau SAR, China; 3 Department of Psychology, De La Salle University, Manila 1004, Philippines

**Keywords:** guanxi, types of strangers, cooperation, public goods dilemma

## Abstract

This study explored people’s estimation of cooperative intention when paired with people with different types of relationships, and the mediating roles of trust and responsibility between guanxi perception and the estimation of cooperative intention. We recruited 398 university students from the Greater Bay Area of China to complete two public goods dilemma experiments. Study 1 manipulated the type of partner to be either family member, classmate, and stranger, representing different types of *guanxi*. Study 2 manipulated the type of partner to be either stranger with intermediary, stranger within ingroup, and complete stranger. In both studies, the mediating roles of trust and responsibility in the relationship between guanxi perception and the estimation of cooperative intention were tested. The results of study 1 showed that the participants’ estimation of cooperative intention with a family member was higher than with acquaintances or with strangers. In study 2, the estimation of cooperative intention with stranger with intermediary was higher than with a stranger within one’s ingroup or with a complete stranger. Multivariate analysis verified the mediating effects. The results are discussed with reference to why Chinese people treat different types of guanxi distinctly, especially to different types of strangers, and how guanxi perception, trust, and responsibility work together to the influence of the estimation of cooperative intention.

## 1. Introduction

Chinese people value *guanxi* (關係, literally, personal connection), which is a culture value of interpersonal relationships. Guanxi is described as “an indigenous Chinese construct defined as an informal particularistic personal connection between two individuals who are bounded by an implicit psychological contract to follow the norm of guanxi, such as maintaining a long-term relationship, mutual commitment, loyalty and obligation” [1]. Numerous studies have shown that guanxi culture influence people’s ideologies [2], trust [3,4], moral judgements [5,6], and cooperative behaviors [7,8]. Most of the previous empirical studies have found that individuals were more willing to cooperate with familiar people on both explicit and implicit levels [9,10]. Guanxi research usually refers to family members, friends, and strangers as a traditional distinction between different personal relationship categories. However, we think that such a sharp distinction is not enough to clarify the essence of guanxi culture. To have a deeper understanding of guanxi culture, this study investigates more diverse relationship categories and study how these differences relate to intentions to cooperate with the target persons.

### 1.1. Guanxi Culture and Traditional Personal Relationship

In the context of guanxi culture, Ho [11,12] underscored ‘relational orientation’ and pointed out that guanxi was more impactful than personal willingness and environmental factors on Chinese people’s social behaviors. Social actions are not determined by personal sentiments, goals, and decisions, but by one’s relationships with other people, and as such, Chinese people are assumed to have a relational self [11]. To support this notion, there is evidence that that for Chinese people, thinking about one’s mother and oneself elicited activity in the same brain region (mPFC) whereas Westerners only used this region to represent the self. The findings provided neuroimaging evidence for Chinese people’s relational selves [13,14,15,16].

Previous empirical research on guanxi usually used the *jia-ren* (family members), *shou-ren* (acquaintances), and *sheng-ren* (strangers) categories proposed by Yang [17], and research on guanxi focused on the difference between family member/acquaintance and strangers. For example, Chinese participants showed more cooperative tendency when the partner was a friend compared to when the partner was a stranger [10]. Chinese people also tend to favor friends over strangers [5], and they give significantly lighter punishment to friends for their deception than to strangers [18]. IAT studies even suggest that the cooperative tendency was more in a familiar relationship (e.g., friends, roommates) than in a stranger relationship [9]. In general, we hypothesize *Chinese participants’ estimation of cooperative intention with a family member will be greater than with acquaintances, and even greater than with strangers* (Hypothesis 1).

Explanations on the effects of *guanxi* focus on the distinction of social distance [10,19,20], which referred to “the extent to which people experience a sense of familiarity (nearness and intimacy) or unfamiliarity (farness and difference) between themselves and people belonging to different social, ethnic, occupational, and religious groups from their own” [21]. This explanation emphasizes the effect of closeness and the differences between ingroup and outgroup [10]. However, we do not think that the distinction between different types of guanxi can be explained simply by closeness and intimacy. One of the most important principles of Chinese people’s treatment of their families is responsibility and obligation. Hwang [22] stated, “According to this rule (i.e., need rule), every member should do his best for the family, and the family will in turn supply him the resources necessary for living” (p. 950). The need rule implies that even if the family is not so emotionally close to each other, one has to consider the needs of the family. Regarding acquaintances, the interactions obey the *renqing* (人情, affect and favor) rule, which is a variant of the equality rule that is tightly bound up with ideas of reciprocity. *Renqing* is the affect shared by people and it motivates them to do favors for each other [22]. As Luo [23] mentioned, “when Chinese people weave their *guanxi* network, they also weave a web of *renqing* obligations. While enjoying the benefits of a connections network, they also take on a reciprocal obligation which must be ‘repaid’ in the future” (p. 53). It shows that the interaction between acquaintances should also consider the responsibility. This is probably the reason why the distinction between different types of guanxi cannot be explained only by social distance; the types of obligations towards other people vary even among people with the same social distance. For example, people will help distant relatives, not because of their close social distance, but because of the perceived obligations towards relatives.

### 1.2. Types of Strangers

The emphasis on social rules of obligation in Chinese guanxi culture is also reflected in that Chinese cultural presupposition that gives precedence to relationships over individuals [24]. Consider this example of a doctor giving special attention to a patient in three different social situations: In situation a, the patient was special (e.g., the symptom was special, or he/she was very poor or very kind), but in situation b, the doctor knew the patient personally. In situation c, the patient was introduced by the doctor’s good friend (or an important guanxi). In the last case, even though the doctor did not know the patient personally and that the patient was not special, the doctor took care of the patient, because the doctor gave face (面子, *mianzi* in Chinese) to his/her good friend. In situation c, what mattered was that the patient is the friend of the doctor’s friend, and that there are social obligations attendant to that type of indirect relationship (see the example in Table 1).

We think that maybe people also classify and treat strangers differently because of the different social obligations related to specific identities and relationships, and not just social distance. In the present investigation, in addition to the typical relationship categories studied in guanxi research, we distinguish three types of strangers: Type 1, stranger with intermediary (e.g., A and B do not know each other, but A and B have a common friend M); Type 2, stranger within ingroup, or strangers who belong to one social group (e.g., A and B do not know each other, but they are schoolfellows); Type 3, complete strangers (i.e., A and B do not have any connections at all).

We assume that among these three types of strangers, people will have more willingness to cooperate with stranger with intermediary, followed by stranger within ingroup, and finally with a complete stranger. This is because for the stranger with intermediary, there is a significant person between the stranger and the client, who plays a very important connecting role in the interaction. In the example in Table 1, *renqing* rule demands that the doctor should take care of his friend’s friend. This is a manifestation of “responsibility” to his friends. As for the stranger within an ingroup, there is no direct responsibility between them. The willingness to cooperate with stranger within ingroup may depend on their sense of belonging to their ingroup, which is a more abstract connection compared to the connection mediated by an intermediary. To summarize, we propose that we will not only find differences between family members, acquaintances, and strangers, but even also among strangers. Therefore, we hypothesize that *Chinese participants’ estimation of cooperative intention with stranger with intermediary will be greater than with a stranger within an ingroup, and then even greater than with complete stranger* (Hypothesis 2).

### 1.3. Guanxi Perception and the Mediation of Trust and Obligation

Chinese people value the identity of the person with whom they are interacting as the identity may inform the perception of intimacy, stability, duration, and importance of the relationship and their attendant relational obligations [22,25]. We refer to these perceptions as guanxi perception. People’s distinction of guanxi types is the result of comprehensive consideration of their guanxi perception. For example, relationships with family members are assessed as intimate, stable, and important, and the relationship with them would be continuous. However, relationships with strangers with intermediaries might be perceived as unstable, noncontinuous, but medium intimate and important, because of the conditional role of the intermediary. Relationships with complete strangers are perceived to be the weakest in all of the four dimensions. Therefore, in this study, we use guanxi perception to measure the strength and quality of a relationship. We assume that people’s estimation of cooperative intention is based on their judgements on the guanxi perception of a relationship.

We also propose that responsibility is another mediator between guanxi perception and cooperative intention. Stronger guanxi perception implies stronger sense of responsibility for the relationship. The renowned Chinese sociologist Fei Xiaotong compared Chinese guanxi to the ripple caused by a stone. In his analogy, each person stood in the center of the ripples as the stone was thrown into water. The ripples were networks connecting people to each other. He called it diversity-orderly structure. In the diversity-orderly structure, “from the most inner ripple to the most outer one, obligations could vary on the level of value and cost… If people do not behave accordingly, they would face social pressure and be regarded as unethical” [26]. In other words, guanxi perception may positively correlate with cooperative intention, through the mediating effect of responsibility.

Finally, trust is a mediator between guanxi perception and the estimation of cooperative intention. Previous studies indicated that trust is one of the major causes of cooperative behaviors [27,28]. However, there are cultural differences in how trust in experienced. Westerners’ trust was mostly cognitive-based trust whereas Easterners’ trust was mostly affect-based trust [29]. Scholars further found that Westerners tend to evaluate trustworthy by estimating individual’s ability and calculating wastage by hypothesizing dishonesty. Whereas Easterners predict trustworthy more depending on context [30]. Additionally, one of the primary contextual factors is guanxi perception. According to the characteristic of affect-based trust, it is possible that the stronger guanxi perception, the more they perceive the partner to be trustworthy, which then relates to higher cooperative intention. In general, we propose the hypothesis that: *Guanxi perception will positively correlate with the estimation of cooperative intention, which will be mediated by trust and responsibility* (Hypothesis 3).

### 1.4. Current Study

**Figure 1:**
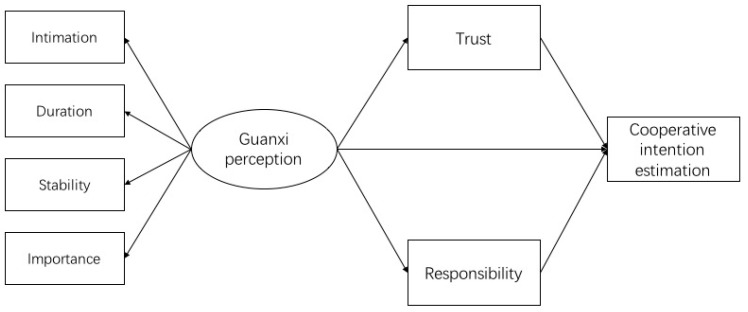
Proposed model of indirect relationship between guanxi perception and estimation of cooperative intention.

Previous guanxi studies only investigated differences between traditional personal relationships, and usually explained the differences in terms of social distance. However, such distinctions among the traditional relationships are not enough to clarify the essence of guanxi culture, and social distance is not enough to explain Chinese people’s distinct treatment of guanxi. In this investigation, we conducted two studies to explore the influence of guanxi perception on people’s cooperative intention and the mediating role of trust and responsibility. In these two studies, we manipulated the guanxi type by traditional personal relationship. We used a projective paradigm to ask participants to assess the cooperative intention of the characters in vignettes (the experimental materials) instead of their own intention. In study 1, participants completed the task three times with the relationship between the character and the partner in materials was either a (1) family member, (2) acquaintance, or (3) stranger. In study 2, the relationships were between the character and a stranger, but different types of strangers, which will be described later. In both studies we explored the effects of qualitative differences in the relationships of the actors, with study 2 making further differentiations within one of the types of relationships in study 1. Aside from comparing whether guanxi perceptions and the estimation of cooperative intentions vary across the different types of relationships, we further tested the mediating roles of trust and obligation in the relationship between guanxi perception and the estimation of cooperative intention in both two studies (please see the proposed model in Figure 1).

## 2. Study 1

### 2.1. Method

#### 2.1.1. Participants

The a priori power analysis (effect size *f* = 0.25) revealed that we needed at least 86 participants in total to have adequate power (1 − *β* = 0.95). We recruited 201 university students (57.7% female) from Guangdong–Hong Kong–Macao Greater Bay Area (31.8% from Guangdong, 34.8% from HK, 33.3% from Macau) to complete a within-subject study. Ages ranged from 17 to 23 years old (*M* = 19.70, *SD* = 1.25). Data were collected by using one of the most popular questionnaire websites in China, named WenJuanXing (www.wjx.cn). Only those who accepted the informed consent form would continue to answer the questions. Each participant who completed the questionnaire received a payment of CNY 15 (approximately USD 2.34).

#### 2.1.2. Procedure and Materials

We set a two-player public goods game as the scenario. The participants read the instruction:

You will read some stories about A is attending an investment game. The token in the game can be exchanged to cash. There are three rounds. Each round includes three subactivities. In each round A will play with different partner.

At the beginning of each round, instruction told the participants that “*In this round, A will play with [his/her family member/classmate/a stranger]*”, with the relationship randomly varied across the three rounds. First, the participants were asked to rate their guanxi perception of the relationship between A and the partner by answering “*How intimate/lasting/stable/important do you think A and the [family member/classmate/stranger?]*”. Participants assessed from 1 (*very distant/totally temporary/very unstable/very unimportant*) to 6 (*very intimate/totally long-term/very stable/very important*). The mean of these four items represented the strength of guanxi perception.

Then, the participants read the scenarios. The statement of subactivity 1 was “A has 30 tokens as the initial investment capital. He could invest any amount into a public account, and the capital in public account will be appreciated. After the appreciation, [family member/classmate/stranger] WILL DECIDE how to assign the money”. The participants were required to answer the question: How much do you think that A will invest to the public account? The higher the amount means the participant indicates higher trust on the partner.

Subactivity 2 was “There are 50 tokens in the public account. A has rights to assign the money”. The participants answered the question: “How much do you think A SHOULD assign to the [family member/classmate/stranger]?” The higher the amount means the participant indicates higher obligation to the partner.

Subactivity 3 was “Both of A and the family member/classmate/stranger have 100 tokens to invest. They could invest any amount into the public account and it will appreciate 1.5 times. After the appreciation, A and the family member/classmate/stranger will split the money”. To help the participants understand the game well, two examples (A invests 100/0 tokens and the partner invests 0/100, then A gains 75/175 tokens and the partner gains 175/75 tokens) were provided in this subactivity. The participants answered the question: “How much do you think that A will invest to the public account?” When answering this question, the participants should understand that if A invested more than the partner, A would earn less than the partner and even face a loss. Therefore, the more A invested, the riskier he/she was. In this case, a higher amount means the participant indicates a higher cooperative intention.

### 2.2. Results and Discussion

#### 2.2.1. Estimation of Cooperative Intention between Traditional Types of Guanxi

The descriptive statistics of the key variables are showed in Table 2 and the correlation results are showed in Table 3. The data were analyzed in the ANOVA with repeated measures on cooperative intention. Guanxi types (family member vs. classmate vs. stranger) was a within subject variable.

For the guanxi perception, the Mauchly’s test in the ANOVA indicated that the assumption of sphericity was violated, *χ*^2^ (2) = 35.09, *p* < 0.001; therefore, degrees of freedom were corrected using Huynh–Feldt estimates of sphericity (ε = 0.87). The results indicated that the main effect of guanxi types on guanxi perception was significant, *F* (1.74, 347.05) = 358.89, *p* < 0.001, *η*^2^ = 0.64. People perceived that family members were stronger than acquaintances and strangers at the 0.001 level. The manipulation of guanxi types was successful.

For the cooperative intention, the Mauchly’s test in the ANOVA indicated that the assumption of sphericity was violated, *χ*^2^ (2) = 18.15, *p* < 0.05; therefore, degrees of freedom were corrected using Huynh–Feldt estimates of sphericity (ε = 0.93). The results indicated that the main effect of guanxi types on estimation of cooperative intention was significant, *F* (1.86, 371.18) = 87.56, *p* < 0.001, *η*^2^ = 0.31. Post hoc comparisons revealed that participants’ estimation of cooperative intention was highest when the interaction partner was a family member (65.81 ± 28.37), and it was lowest when the partner was a stranger (36.15 ± 27.31). All pairwise comparison differences were significant at *p* < 0.001. Hypothesis 1 was supported.

#### 2.2.2. Pathways to the Estimation of Cooperative Intention

**Figure 2:**
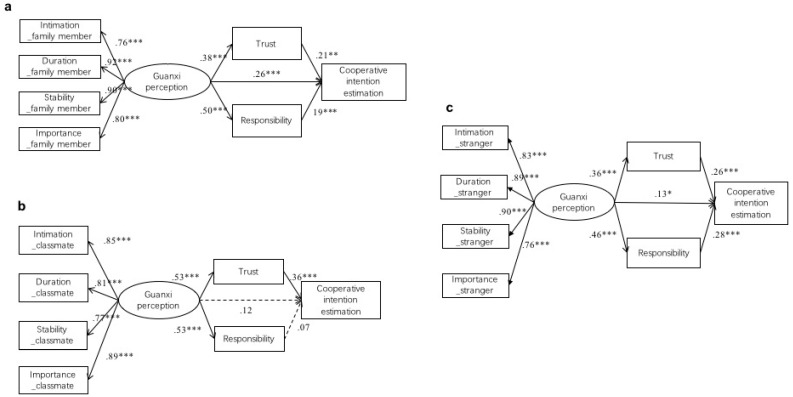
Model of indirect relationship between guanxi perception and estimation of cooperative intention in study 1, in which the partner was (**a**) family member, (**b**) classmate, and (**c**) stranger. Broken lines indicate non-significant paths. * *p* < 0.05, ** *p* < 0.01, *** *p* < 0.001.

To figure out what factors affected the different evaluations of cooperative intention between the types of guanxi, three path analyses were conducted using AMOS 23.0 to test the hypothesized model (Figure 1), one model for each partner condition. For family member, the results indicated a good model fit (*χ*^2^ = 19.496, *p* = 0.021, *χ*^2^*/df* = 2.166, GFI = 0.973, TLI = 0.980, CFI = 0.987, RMSEA = 0.076). As shown in Figure 2a, the direct effect of guanxi perception on estimation of cooperative intention was significant, and the relationship was partially mediated by trust and obligation. Indirect effects were examined using bootstrapping (95% bias-corrected confidence interval, 10,000 samples). Results showed that guanxi perception had a significant indirect effect on the estimation of cooperative intention through trust and obligation with a point estimate of *β* = 0.18 (95% CI: 0.10–0.28).

For the interaction with a classmate, results indicated a good model fit (*χ*^2^ = 6.776, *p* > 0.05, *χ*^2^*/df* = 0.678, GFI = 0.991, TLI = 0.966, CFI = 1.000, RMSEA < 0.001, 90% CI: 0.000–0.055). As shown in Figure 2b, the direct effect of guanxi perception on the estimation of cooperative intention was not significant, and the relationship was only mediated by trust. Bootstrapping results showed that guanxi perception had a significant indirect effect on the estimation of cooperative intention mainly through trust with a point estimate of *β* = 0.28 (95% CI: 0.18–0.41).

For the interaction with a stranger, the results indicated a good model fit (*χ*^2^ = 9.236, *p* > 0.05, *χ*^2^*/df* = 0.84, GFI = 0.987, TLI = 1.005, CFI = 1.000, RMSEA < 0.001, 90% CI: 0.000–0.065). As shown in Figure 2c, the direct effect of guanxi perception on the estimation of cooperative intention was significant, and the relationship was partially mediated by trust and obligation. Indirect effects were examined using bootstrapping (95% bias-corrected confidence interval, 10,000 samples). Results showed that guanxi perception had a significant indirect effect on the estimation of cooperative intention through trust and obligation with a point estimate of *β* = 0.21 (95% CI: 0.11–0.32).

The pathways to cooperative intention were similar when the partner was family member and stranger. In both two models (i.e., Figure 2a,c), guanxi perception predicted the estimation of cooperative intention partially through both trust and obligation. However, when the partner was classmate, guanxi perception only affect the estimation of cooperative intention through trust. It might suggest that for acquaintances, trust was the most important element for the relation quality.

## 3. Study 2

In study 1, we primarily tested participants’ different evaluations on cooperative intention between traditional guanxi types and explored the pathways from guanxi perception to the estimation of cooperative intention. In study 2, we extend the investigation to more specific relationship types that are not typically referred to in most guanxi research. In particular, we examined different types of relationships with strangers as described in the introduction: stranger with intermediary, stranger within ingroup, and a complete stranger. According to the inference based on guanxi culture that we discussed in the introduction, we assumed that the different evaluations on cooperative intention would also exist among different types of strangers. In study 2, we used the similar procedure and experimental materials to study 1 to test the hypotheses. The only change was that the partners in the scenarios of study 2 were different types of strangers.

### 3.1. Method

#### 3.1.1. Participants

The a priori power analysis (effect size *f* = 0.25) requested at least 86 participants in total to have adequate power (1 − *β* = 0.95). We recruited 197 university students (45.2% female) from Guangdong–Hong Kong–Macao Greater Bay Area (34% from Guangdong, 33% from HK, 33% from Macau) to complete a within-subject study. Ages ranged from 17 to 25 years old (*M* = 19.87, *SD* = 1.79). The data collecting process was the same as study 1.

#### 3.1.2. Measures

Compared with study 1, the only change was the roles of partners. Instead of family member, classmate, and stranger, the partners in study 2 were three types of strangers. For stranger with intermediary, the instruction was “*At this round, coincidentally, the strange partner randomly assigned to A is a friend of M, who is also A’s friend*”. For stranger within ingroup, the instruction was “*At this round, from the background information, A knows that the strange partner randomly assigned to is a schoolfellow*”. For the complete stranger, the instruction was “*At this round, A plays the game with a strange partner who is assigned randomly*”. The subactivities and questions were same as study 1.

### 3.2. Results and Discussion

#### 3.2.1. Estimations of Cooperative Intention between Types of Strangers

The descriptive statistics of the key variables are in Table 2 and the correlation results are showed in Table 3. The results were also analyzed in the ANOVA with repeated measures on cooperative intention. Type of strangers was within subject variable.

For the guanxi perception, the Mauchly’s test in the ANOVA indicated that the assumption of sphericity was violated, *χ*^2^ (2) = 9.19, *p* = 0.01; therefore, degrees of freedom were corrected using Huynh–Feldt estimates of sphericity (ε = 0.97). The results indicated that the main effect of guanxi types on guanxi perception was significant, *F* (1.93, 378.35) = 144.17, *p* < 0.001, *η*^2^ = 0.42. People perceived family members were stronger than acquaintances and strangers at the 0.001 level. The manipulation of stranger types was successful.

For the estimation of cooperative intention, results indicated that the main effect of stranger types was significant, *F* (2, 392) = 41.33, *p* < 0.001, *η*^2^ = 0.17. Post hoc comparisons revealed that, participants’ estimation of cooperative intention was highest when with the stranger with intermediary (i.e., M’s friend; 51.25 ± 28.35), and was lowest when with complete stranger (34.69 ± 28.60). All pairwise comparison differences were significant at *p* < 0.001. Hypothesis 2 was supported.

#### 3.2.2. Pathways to Cooperative Intention

**Figure 3:**
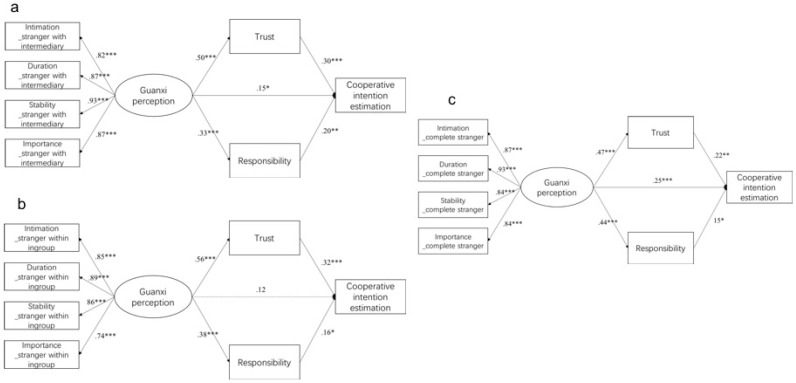
Model of indirect relationship between guanxi perception and estimation of cooperative intention in study 2, in which the partner was (**a**) stranger with intermediary (e.g., friend’s friend), (**b**) stranger within ingroup (e.g., schoolfellow), and (**c**) complete stranger. Broken lines indicate non-significant paths. * *p* < 0.05, ** *p* < 0.01, *** *p* < 0.001.

For stranger with intermediary, the results indicated a good model fit (*χ*^2^ = 10.669, *p* > 0.05, *χ*^2^*/df* = 0.97, GFI = 0.985, TLI = 1.001, CFI= 1.000, and RMSEA < 0.001, 90% CI: 0.000–0.073). As shown in Figure 3a, the direct effect of guanxi perception on the estimation of cooperative intention was significant, and the relationship was partially mediated by trust and obligation. Indirect effects were examined using bootstrapping (95% bias-corrected confidence interval, 10,000 samples). Results showed that guanxi perception had a significant indirect effect on the estimation of cooperative intention through trust and obligation with a point estimate of *β* = 0.22 (95% CI: 0.12–0.34).

For stranger within ingroup, results indicated a good model fit (*χ*^2^ = 11.939, *p* > 0.05, *χ*^2^*/df* = 1.085, GFI = 0.984, TLI = 0.997, CFI = 0.999, and RMSEA = 0.021). As shown in Figure 3b, the direct effect of guanxi perception on the estimation of cooperative intention was not significant, but the relationship was mediated by both trust and obligation. Bootstrapping results showed that guanxi perception had a significant indirect effect on the estimation of cooperative intention mainly through trust with a point estimate of *β* = 0.24 (95% CI: 0.14–0.36).

For the complete stranger, the results indicated a good model fit (*χ*^2^ = 6.520, *p* > 0.05, *χ*^2^*/df* = 0.593, GFI = 0.991, TLI = 1.011, CFI = 0.966, and RMSEA < 0.001, 90% CI: 0.000–0.044). As shown in Figure 3c, the direct effect of guanxi perception on the estimation of cooperative intention was significant, and the relationship was partially mediated by trust and obligation. Indirect effects were examined using bootstrapping (95% bias-corrected confidence interval, 10,000 samples). Results showed that guanxi perception had a significant indirect effect on the estimation of cooperative intention through trust and obligation with a point estimate of *β* = 0.17 (95% CI: 0.09–0.28).

## 4. Discussion

This investigation explored how people evaluated people’s cooperative intentions in tasks that involve persons of different guanxi types and also considered the influence of guanxi perception and the mediating effects of trust and obligation. Consistent with many previous studies, the results of study 1 and 2 confirmed the distinguish between family members, acquaintances, and strangers as shown by the significant differences in the estimation of cooperative intention depending on the type of relationship between the cooperating partners. The verification of hypothesis 1 and 2 indicates Chinese people’s apparent differentiation between different guanxi types. However, the difference between treating acquaintances and strangers in Western society does not seem to be that obvious. For example, some research showed that American participants treated friends and strangers equally [31]. Low-income Mexican villages gave more money to family members than to community members and strangers in the dictator game, but there was no significant difference between the latter two groups [18].

More interestingly, our experiment indicated that Chinese people not only distinguish acquaintances and strangers, but even distinguish different types of strangers. Specifically, people believed that a person would show more cooperative intention when the partner was his/her friend’s friend, than when the partner and himself/herself belonged to a same group than when the partner was a complete stranger. We proposed that the phenomenon could be explained by the effect of trust and obligation, and our results verified the proposition. The verification of hypothesis 3 indicated that the distinct cooperation degree of the five types of guanxi reflected the various guanxi perceptions (intimacy, duration, stability, and importance) of these guanxi types through the mediations of trust and obligation.

An innovative contribution of the current study is that we categorized the types of strangers, and the significant differences between stranger with intermediary and stranger within ingroup are especially reflective of the essence of guanxi. Since the partners did not know each other per se, no matter what kinds of strangers, if people made judgements according to cognitive-based trust, they should treat all the strangers the same. Nevertheless, we found people’s distinct treatment to different kinds of strangers, depending on what we presume are different degrees of intimacy, duration, stability, and importance even if these relationship dimensions are low given that strangers are involved. As expected, when a person played games with someone knowing his/her friend, even he/she did not know the partner personally, people assumed he/she would express more cooperative intention than other kinds of strange partners. Stranger with intermediary might actually involve a “partial stranger” because people will consider the intermediary when they make their decision. The presence of an intermediary not only bridges the intimacy of the two parties, but also increases the perception of stability, duration, and importance. This perception is largely mixed with the perception of the intermediary. Although the guanxi perception of stranger within ingroup was weaker than that of stranger with intermediary, people still gave a higher estimation of cooperative intention than to complete stranger. This result aptly reflects the Chinese guanxi culture, where “relationships come to precede individuals”. In fact, people’s guanxi perception of the stranger within ingroup is not with a specific strange alumnus or fellow villager, but with the ingroup. It is the ingroup instead of a person makes them feel a certain degree of intimacy, duration, stability, and importance.

From the results of path analyses we know that when people estimated cooperative intention, they actually assessed the quality of guanxi according to the guanxi perception. Additionally, the effect of guanxi perception on estimation of cooperative intention mainly mediated by trust and responsibility. The strong connection between trust and cooperation has been confirmed [26,32]. Additionally, the mediating role of trust in our research further suggests that Chinese people’s trust is affect-based trust. The more people perceive the relationship to be of high quality, the more they perceive the partner to be trustworthy—even if the partner is some kind of stranger. Especially for acquaintances, the effect of guanxi perception on estimation of cooperative intention was only mediated by trust. Acquaintances are neither as high a level of guanxi perception as a family member, nor as low a level of guanxi perception as a stranger. We suggest that the identity of the acquaintance maybe not enough to allow people to easily make a decision about cooperation. People may have to think through their perceived trust of the ingroup stranger to decide on the cooperative intention.

On the other hand, the mediation of responsibility may manifest in righteousness (義, *yi*), which refers to various aspects in different types of guanxi. For family members, righteousness means filial duty and loyalty. For friends or acquaintances, righteousness parallels the norm of reciprocity and equality. This could explain why people think they should assign more money to family member than to the classmate. Filial duty and loyalty encourage family members to dedicate to each other. Moreover, in the background of guanxi culture, individuals would think that family members belong to each other. Therefore, people think they should, and they are willing to, assign money to the family member. For stranger with intermediary, people believe that we should “take care of” friend’s important others. To some extent it could be understood as a kind of responsibility. This is one of the connotations of *renqing*. For stranger within ingroup, righteousness means solidarity. When people perceive the ingroup, the collectivist culture would remind them the norm of solidarity. As a result, people assess higher cooperative intention with the stranger within ingroup than with the complete stranger.

This study had some limitations. Although we chose university students with the idea that “they represent the younger generation in China who are influenced by Western culture”, they might have limited social experiences and understandings on guanxi norms and practices. Future studies could recruit community samples to strengthen the validity of the findings. Second, the present study has focused on examining the mediating role of trust and responsibility; however, there might be other important mediating factors. For example, an unknown leader and an unknown citizen may have various degrees of importance because of their various social status, especially for those who believe that guanxi culture is essential and required in Chinese communities. Researchers can also explore other forms of relationships among strangers, considering power status, for example. Additionally, the influence of guanxi perception on cooperative intention might not mediated by trust and responsibility. Maybe pragmatic and practical considerations are more important.

While the limitations of the current study are worth considering, we believe that our results point to small but important steps forward by providing evidence of the distinction between different types of strangers. With the development of society, Chinese people’s interpersonal interaction patterns have also undergone some changes. The types of daily interactions may no longer be limited to family members and acquaintances as the increased population mobility. In Chinese big cities (e.g., the Greater Bay Area of China cities where our participants came from), people are becoming more and more engaged with and interacting with strangers. Our results show that regarding trust, responsibility, and cooperative intention, people treat strangers distinctly because of their different identities. It is helpful to have further insight into interpersonal interactions, especially stranger interactions, in contemporary Chinese societies under the context of guanxi culture. Moreover, we chose university students from the Greater Bay Area including Hong Kong and Macau as participants because this is a group of youth who live in some of the most economically developed cities in China, receive higher education, and have also received many Western educational thoughts. However, we still see that they treat different types of guanxi with distinction. This implies that Chinese guanxi culture still has a profound influence on Chinese societies. Despite the rapid changes that are taking place in China, guanxi culture is likely to remain an important aspect of Chinese society for years to come.

## 5. Conclusions

To sum up, findings in this study showed that Chinese people estimated the cooperative intention with family members as higher than acquaintance or strangers. Moreover, their estimation of cooperative intention with a stranger with an intermediary was higher than a stranger within ingroup or with a complete stranger. Guanxi perception, involving the perception of intimacy, duration, stability, and importance of the guanxi, influenced estimation of cooperative intention positively, through the mediations of trust and responsibility. In these two studies, we provide conceptual and empirical extensions to how guanxi principles relate to more varied forms of interpersonal interactions in fast changing Chinese societies.

## Figures and Tables

**Table 1:** Examples of different types of guanxi between a doctor and the patient who is paid special attention.

	Guanxi between the Doctor and the Patient	Identity of the Patient	The Doctor’s Possible Inner Thoughts
Situation a	complete stranger	Just a patient	“The patient/symptom is special, I’m going to be careful.”
Situation b	acquaintance	A friend of the doctor	“This is my good friend, I’m going to be careful.”
Situation c	stranger with intermediary	A patient and a friend of the doctor’s friend	“This is XX’s friend, I should be careful.”

**Table 2:** Descriptive statistics for key variables in study 1 and study 2.

		Cooperative Intention Estimation (Max 100)	Trust (Max 30)	Responsibility (Max 50)	Guanxi Perception			
					Intimation	Duration	Stability	Importance
Study 1	Family member	65.81 (±28.37)	19.96 (±7.36)	28.31 (±10.14)	5.11 (±0.97)	5.11 (±1.04)	5.00 (±1.08)	5.16 (±0.97)
	Classmate	52.85 (±28.10)	16.06 (±6.37)	24.55 (±9.22)	4.38 (±1.00)	4.11 (±1.12)	4.19 (±1.18)	4.29 (±1.17)
	Stranger	36.15 (±27.31)	12.27 (±7.27)	20.70 (±10.13)	2.27 (±1.32)	2.52 (±1.35)	2.56 (±1.40)	2.84 (±1.47)
Study 2	Stranger with intermediary	51.25 (±28.35)	15.35 (±6.21)	24.15 (±8.52)	3.66 (±1.12)	3.59 (±1.16)	3.57 (±1.17)	3.47 (±1.24)
	Stranger within ingroup	43.22 (±27.23)	12.84 (±5.95)	21.69 (±8.75)	3.04 (±1.08)	3.01 (±1.08)	3.00 (±1.09)	2.98 (±1.11)
	Complete stranger	34.69 (±28.60)	10.83 (±6.52)	19.94 (±9.98)	2.14 (±1.22)	2.36 (±1.26)	2.40 (±1.29)	2.44 (±1.33)

**Table 3:** Correlations between key variables in study 1 and study 2.

			1 Cooperative Intention Estimation	2 Trust	3 Responsibility	4 Intimation	5 Duration	6 Stability
	Family member	2	0.42 ***					
		3	0.46 ***	0.54 ***				
		4	0.47 ***	0.46 ***	0.39 ***			
		5	0.39 ***	0.41 ***	0.34 ***	0.68 ***		
		6	0.39 ***	0.42 ***	0.35 ***	0.73 ***	0.84 ***	
		7	0.38 ***	0.43 ***	0.38 ***	0.76 ***	0.76 ***	0.72 ***
	Classmate	2	0.50 ***					
		3	0.42 ***	0.70 ***				
Study 1		4	0.28 ***	0.48 ***	0.46 ***			
		5	0.26 ***	0.47 ***	0.47 ***	0.66 ***		
		6	0.33 ***	0.50 ***	0.46 ***	0.71 ***	0.77 ***	
		7	0.30 ***	0.47 ***	0.42 ***	0.77 ***	0.70 ***	0.69 ***
	Stranger	2	0.47 ***					
		3	0.51 ***	0.59 ***				
		4	0.25 ***	0.26 ***	0.34 ***			
		5	0.24 **	0.26 ***	0.40 ***	0.72 ***		
		6	0.30 ***	0.25 ***	0.35 ***	0.77 ***	0.77 ***	
		7	0.28 ***	0.23 **	0.35 ***	0.63 ***	0.67 ***	0.67 ***
Study 2	Stranger with intermediary	2	0.49 **					
		3	0.41 **	0.52 **				
		4	0.33 **	0.35 **	0.26 **			
		5	0.32 **	0.44 **	0.25 **	0.73 **		
		6	0.34 **	0.48 **	0.31 **	0.75 **	0.80 **	
		7	0.33 *	0.44 **	0.29 **	0.71 **	0.74 **	0.81 **
	Stranger within ingroup	2	0.48 **					
		3	0.38 **	0.53 **				
		4	0.27 **	0.44 **	0.33 **			
		5	0.32 **	0.50 **	0.33 **	0.76 **		
		6	0.32 **	0.50 **	0.34 **	0.73 **	0.78 **	
		7	0.36 **	0.47 **	0.28 **	0.65 **	0.65 **	0.62 **
	Complete stranger	2	0.42 **					
		3	0.38 **	0.52 **				
		4	0.37 **	0.42 **	0.39 **			
		5	0.42 **	0.42 **	0.41 **	0.81 **		
		6	0.36 **	0.40 **	0.40 **	0.74 **	0.77 **	
		7	0.32 **	0.41 **	0.35 **	0.72 **	0.79 **	0.71 **

Note. * *p* < 0.05, ** *p* < 0.01, *** *p* < 0.001.

## Data Availability

Not applicable.
